# Association of recent respiratory illness and influenza with acute myocardial infarction among the Bangladeshi population: A case–control study

**DOI:** 10.1017/S0950268823001863

**Published:** 2023-11-30

**Authors:** Mohammad Abdul Aleem, C. Raina Macintyre, Bayzidur Rahman, A. K. M. Monwarul Islam, Zubair Akhtar, Fahmida Chowdhury, Firdausi Qadri, Abrar Ahmad Chughtai

**Affiliations:** 1 School of Population Health, UNSW Medicine, The University of New South Wales, Sydney, NSW, Australia; 2 Program for Emerging Infections, Infectious Diseases Division, International Centre for Diarrhoeal Disease Research, Bangladesh (icddr, b), Dhaka, Bangladesh; 3 Biosecurity Program, The Kirby Institute, The University of New South Wales, Sydney, NSW, Australia; 4 Department of Cardiology, National Institute of Cardiovascular Diseases (NICVD), Dhaka, Bangladesh; 5 Respiratory and Enteric Infections, Infectious Diseases Division, International Centre for Diarrhoeal Disease Research, Bangladesh (ICDDR, B), Dhaka, Bangladesh

**Keywords:** acute cardiovascular events, acute myocardial infarction, acute respiratory illness, case–control study, influenza

## Abstract

Current evidence suggests that recent acute respiratory infections and seasonal influenza may precipitate acute myocardial infarction (AMI). This study examined the potential link between recent clinical respiratory illness (CRI) and influenza, and AMI in Bangladesh. Conducted during the 2018 influenza season at a Dhaka tertiary-level cardiovascular (CV) hospital, it included 150 AMI cases and two control groups: 44 hospitalized cardiac patients without AMI and 90 healthy individuals. Participants were matched by gender and age groups. The study focused on self-reported CRI and laboratory-confirmed influenza ascertained via quantitative real-time reverse transcription polymerase chain reaction (qRT-PCR) within the preceding week, analyzed using multivariable logistic regression. Results showed that cases reported CRI, significantly more frequently than healthy controls (27.3% vs. 13.3%, adjusted odds ratio (aOR): 2.21; 95% confidence interval (CI): 1.05–4.06), although this was not significantly different from all controls (27.3% vs. 22.4%; aOR: 1.19; 95% CI: 0.65–2.18). Influenza rates were insignificantly higher among cases than controls. The study suggests that recent respiratory illnesses may precede AMI onset among Bangladeshi patients. Infection prevention and control practices, as well as the uptake of the influenza vaccine, may be advocated for patients at high risk of acute CV events.

## Introduction

Cardiovascular (CV) diseases (CVDs) are the leading cause of death globally [1, 2], and their burden has nearly doubled since 1990. According to an estimate, around 18.1 million individuals died from CVDs in 2019 globally, with nearly half of the deaths attributed to ischaemic heart diseases, such as acute myocardial infarction (AMI) [2]. Similarly, the seasonal influenza virus accounts for more than 5 million acute respiratory infection (ARI) hospitalizations and up to more than 600,000 deaths every year among adults [3]. Moreover, the 2009 pandemic H1N1 influenza is thought to have contributed to an additional 83,300 CV deaths globally, indicating an unrecognized burden due to influenza-associated adverse CV outcomes [4].

Current epidemiological and experimental evidence suggests that recent ARI due to influenza or other respiratory viruses may increase the risk of adverse acute CV events, including AMI, among vulnerable adults [5, 6]. Pooled estimates from a recent meta-analysis of self-controlled case-series studies showed an increased risk of AMI if ARI was reported due to influenza within the past three days Incidence Rate Ratio (IRR: 5.79; 95% confidence interval (CI): 3.59–9.38) [7]. Additionally, estimates from the meta-analysis of case–control studies showed a twofold risk of AMI after the onset of recent ARI and influenza (pooled OR: 2.01; 95% CI: 1.47 to 2.76) [8]. Furthermore, observational studies showed that vulnerable adults who received the influenza vaccine were less likely to experience recurring adverse CV events, such as AMI, when compared to unvaccinated adults [9, 10]. A large-scale randomized clinical trial, Influenza Vaccination After Myocardial Infarction (IAMI), reported a 40% reduction in CV deaths among patients who received influenza vaccines early after the onset of AMI [9]. According to a recent meta-analysis conducted by Jaiswal et al., which included both observational studies and trials, it was found that influenza vaccination significantly decreased major adverse CV events (MACEs), all-cause mortality, CV mortality, and myocardial infarction among high-risk populations [10]. Further evidence from time-series studies revealed an upward trend in epidemics of AMI-associated hospitalizations and deaths during the winter months of the influenza season, mirroring the pattern of influenza activity [11, 12]. This indirect evidence linking influenza and ARI with AMI provided valuable insights and inspired further research to generate more compelling direct evidence from case-series and case–control studies to strengthen the hypothesis.

Currently, the majority of the global burden of chronic underlying comorbid CV conditions [13] and the prevalence of associated CV risk factors [14] such as smoking, atherogenic diet, and less physical activity are among the populations of low- to middle-income countries, including Bangladesh [15]. Moreover, the onset of adverse acute CV events like AMI is at a relatively younger age among these populations [16]. The national surveillance data suggest that seasonal influenza circulating during the rainy season in Bangladesh may directly contribute to the considerable number of hospitalizations and deaths among at-risk adults and elderly [17–19]. Furthermore, there are poor infection and prevention control practices and gross underutilization of the influenza vaccine among high-risk adults in Bangladesh [20]. Bangladesh is one of the most densely populated countries in the world, which can notably influence the transmission dynamics of respiratory infections such as influenza. Challenges such as air pollution and overcrowding, coupled with limited access to healthcare in such densely populated regions, could potentially exacerbate AMI incidences. Given these circumstances, we speculate that ARI due to influenza or other respiratory viruses could be important contributors to excess AMI incidences during influenza seasons in low- to middle-income settings such as Bangladesh. Currently, there is no national policy or guidelines for the utilization of the influenza vaccine among CV patients in Bangladesh. To date, no studies have explored the potential link between ARI, including influenza, and AMI patients in Bangladesh, which could inform influenza control policies in the country. Such data may help officials explore the potential benefits of immunizing specific subgroups of individuals at high risk of circulatory burden. Policies around the control of influenza prioritizing the influenza vaccination among adults and elderly individuals at high risk of AMI may contribute to mitigation efforts against the burden of hospitalizations and deaths due to influenza-associated AMI in Bangladesh. Furthermore, the integration of data from low-income countries such as Bangladesh is pivotal for an all-encompassing comprehension of this subject matter, specifically for conditions such as influenza and CV diseases, which have a global distribution and influence. Unique insights collected from low-income nations, which are attributed to distinctive healthcare systems, accessibilities, and socio-economic determinants, can significantly influence disease prevalence, management, and outcomes. However, we should acknowledge the potential underrepresentation of research from these nations due to a multitude of factors including, but not limited to, lower research productivity, obstacles in publishing in international academic journals, or an absence of representation in the databases explored. On these accounts, this study aimed to investigate the link between ARI within the past week and confirmed influenza with AMI, in a large tertiary care CV hospital in Bangladesh.

## Methods

### Study design and study population

A case–control study was conducted to determine the association of recent ARI and influenza with AMI at the National Institute of Cardiovascular Diseases in Dhaka, Bangladesh (NICVD), during the 1 May to 31 October 2018 influenza season [17]. In Bangladesh, the rainy season typically lasts from May to October each year, coinciding with the peak of influenza circulation. The description of annual influenza seasons for Bangladesh based on national surveillance data is discussed in detail by Zaman et al. [17]. Bangladesh is the eighth-most populous country in the world, with a population exceeding 165 million, and also among the most densely populated countries in the world [21]. The NICVD is the largest government-run tertiary care teaching hospital exclusively specialized in the management of patients with any cardiac conditions, serving individuals from all regions of the country. However, most of the patients admitted to NICVD, with AMI or other acute cardiac conditions, come from Dhaka, the capital city of Bangladesh, with a population of around 15 million, and from surrounding semi-urban and rural areas.

### Selection of cases and controls

Trained research staff recruited patients aged ≥40 years hospitalized during the 2018 annual influenza season with the diagnosis of AMI from inpatient wards of the cardiology department including coronary care units (CCU) and general cardiology wards of NICVD. Staff followed a standardized protocol to identify and recruit cases with AMI, diagnosed on Electrocardiogram (ECG) findings S and T wave of an electrocardiogram (ST-segment elevation or depression and pathological Q waves) and ischaemic symptoms (chest or arm pain, nausea or vomiting, sweating, and shortness of breath), with either a change in blood level of cardiac biomarkers of myocardial necrosis (typical rise and gradual fall in troponin or more rapid rise and fall in creatine kinase myocardial band (MB)) or coronary artery intervention [22].

To minimize the potential misclassification bias, after reviewing the criteria for the case definition of AMI from hospital records, staff consulted with an attending cardiologist to further ensure the diagnosis of AMI. Cases with a previous history of ischaemic CV events such as acute coronary syndrome or stroke were eligible. The exclusion criteria were cases with a self-reported history of chronic liver impairment, chronic renal impairment, malignancy, and autoimmune disorders; cases unable to provide respiratory samples within 72 h of onset of AMI; and cases not providing consent. Cases travelling from Dhaka or surrounding semi-urban and rural areas were recruited.

The controls were composed of two groups: 1). cardiac controls: cardiac patients aged ≥40 years hospitalized due to any cardiac events other than acute ischaemic events such as AMI and stable or unstable angina and 2). healthy controls: apparently healthy individuals aged ≥40 years. In the initial phase of the study, inpatient cardiac controls were recruited for comparison with the AMI cases. However, to address potential overmatching issues related to the cardiac controls and to minimize the chance of selection bias due to the misclassification of cardiac controls, healthy controls were later included in the study starting from early August 2018. An interim analysis revealed that most of the cardiac controls admitted to NICVD were patients with acute cardiac events. In this group, we observed a higher occurrence of CRI and influenza than expected. Additionally, enrolling eligible cardiac controls for each AMI case became challenging, as more than 70% of inpatients at NICVD were admitted with ischaemic complications including AMI. The cardiac controls included diagnoses such as valvular heart diseases, septal defects, valvular insufficiency, acute hypertension, cardiomyopathy, pericarditis, and pericardial effusion. The healthy controls were recruited from [1] consenting visitors of index AMI cases who were not their blood relatives and [2] visitors of other inpatients in the same wards as the index AMI cases who were not part of this study. Controls were frequency-matched to AMI cases for gender and age group in 5-year age bands. This was implemented to facilitate comparability between the case and control groups. In addition, the controls were recruited during the same week as the cases in the 2018 influenza season to ensure that both the cases and controls had a similar chance of being exposed to acute respiratory illness or circulating influenza. All the enrolled control participants were individuals who travelled from Dhaka or the surrounding semi-urban or rural areas. The exclusion criteria for all controls were a history of acute ischaemic CV events such as AMI, ischaemic stroke, and stable or unstable angina within the prior 12 months of recruitment; suffering from chronic liver or renal impairment, malignancy, and autoimmune disorders; and being unable to provide respiratory samples during enrolment and not providing consent.

Excluding participants with conditions such as chronic liver or renal impairment, malignancy, and autoimmune disorders was primarily to prevent potential confounding factors, including comorbidities, medication usage, and physiological changes, from obscuring the relationship between the primary exposure (recent respiratory illness and influenza) and the observed outcome (AMI). Additionally, this exclusion aimed to address the heightened vulnerability of these groups, who might be more susceptible to respiratory infections such as influenza and experience more severe complications when infected, while also considering that complex medical histories could complicate result interpretation, especially with enduring effects from previous treatments such as chemotherapy. The study employed a case–control ratio of 1:1, indicating that for each case enrolled in the study, the objective was to enrol one control.

### Data collection

Once consent was obtained, the staff reviewed medical records and conducted interviews using a structured questionnaire with participants to gather information on their socio-demographic data, lifestyle, comorbid illnesses, and recent respiratory symptoms. These symptoms included fever, cough, sore throat, runny nose, and difficulty breathing within the past week of the AMI onset or the past week of the recruitment of controls. The staff also took physical measurements of the participants to assess their health status, including clinical examinations and anthropometric measurements.

### Specimen collection and laboratory analysis

Trained staff collected nasopharyngeal and oropharyngeal swabs from all participants including AMI cases and controls, within 72 h of the onset of AMI or of the recruitment of controls and transported them daily in viral transport medium (VTM) at 2–8° C to the Virology Laboratory of the International Centre for Diarrheal Disease Research, Bangladesh (ICDDR,B), Dhaka. Specimen collection within the aforementioned timeframe was conducted to enhance the sensitivity of the study in detecting influenza ribonucleic acid (RNA) before the cessation of viral shedding. The specimens were aliquoted in a bio-safety level 2 (BSL-2) safety cabinet and stored in freezers at or below −70 ° C until analysis. RNA was extracted from the swab specimen, and quantitative real-time reverse transcription polymerase chain reaction (qRT-PCR) was performed using primers and probes specific for influenza A and B viruses provided by the Influenza Division at the CDC. Hemagglutinin subtyping of type A and B viruses was performed to detect subtypes, A/H3, A/H1N1pdm09, B/Victoria, and B/Yamagata. The qRT-PCR was also performed to detect other respiratory viruses including respiratory syncytial virus (RSV), human metapneumovirus (HMPV), human parainfluenza viruses (HPAVs) 1, 2, and 3, and adenovirus. The laboratory used validated protocols [23] and followed standard quality control procedures.

### Exposure measures

The primary explanatory variable was recent clinical respiratory illness (CRI) defined as a self-reported history of ≥2 respiratory symptoms (fever, cough, sore throat, runny nose, and difficulty breathing) within 7 days of the onset of AMI [24, 25] or of the recruitment of controls. The additional exposure measure was qRT-PCR-confirmed influenza A or B in the respiratory swabs.

### Data analysis

### Descriptive analysis of baseline characteristics and respiratory infection rates in cases and controls

Categorical data, such as the frequency of different characteristics, were expressed as percentages, while continuous data, such as height and weight, were expressed as means and standard deviations. Descriptive analyses were performed to compare the baseline characteristics, frequencies, and percentages of CRI and laboratory-confirmed influenza between AMI cases and both all controls and healthy controls. Pearson’s chi-squared test or Fisher’s exact test was used, where appropriate, to ascertain the differences in the proportions of baseline characteristics, CRI, and influenza between the cases and controls. The statistical significance was set at a p-value of 0.05 or lower.

### Logistic regression analyses of respiratory infections in association with AMI

To study the association between recent CRI and laboratory-confirmed influenza with AMI, multivariable logistic regression analyses were performed, adjusting for potential confounders, which included age, gender, tobacco use status, physical activity level, history of hypertension, diabetes, high blood cholesterol, and body mass index (BMI). To express the measures of association, the crude ratio and adjusted odds ratio (aOR) were estimated. All clinically relevant variables and those with a p-value less than 0.25 in the univariable model were included in the initial multivariable base model, and a backward elimination approach was used to sequentially remove the variables. Likelihood-ratio tests were used to determine the goodness of fit of the model after removing each variable. The study employed a sequential elimination process, where the least significant variable was removed based on p-values and the model was refitted, continuing this cycle until all variables approached towards the p-values <0.05 threshold. Co-variates were retained if they were seen to have a confounding effect on the association between CRI and influenza with AMI of 10% or greater. The variance inflation factor (VIF) was also measured for each variable in the initial model to assess multicollinearity.

### Demographic characteristics and respiratory infection rates among cardiac and healthy control participants

In addition, as a part of secondary analyses, which were not the main focus of this study, demographic characteristics were compared between AMI cases and cardiac control participants. Lastly, the frequencies of CRI and influenza were compared between cardiac and healthy control participants using univariate and multivariate analyses.

IBM Statistical Package for the Social Sciences (SPSS) Statistics V.26 was used for all statistical analyses.

### Sample size

Assuming a significance level of 0.05 and a power of 80%, the required sample size was estimated using the proportion of confirmed influenza among cases 20% and controls 10%. Using a two-sided z-test and the difference in proportions (effect size) between cases and controls, the required sample size was calculated to be 90 cases and 90 controls, for a total sample size of 180 participants. The influenza rate estimation was based on national hospital-based influenza surveillance data [17]. We aimed at recruiting 150 cases vs. 150 controls during the 2018 annual influenza season.

## Results

### Demographic characteristics of cases and controls

During the 2018 annual influenza season, a total of 284 participants were recruited, consisting of 150 cases and 134 controls (44 cardiac controls and 90 healthy controls). Demographic and clinical characteristics were compared between cases and all controls (cardiac controls plus healthy controls) and between cases and healthy controls in Table 1. Overall, the demographic and clinical features were comparable between cases and controls, except for tobacco use. The rate of tobacco use was significantly higher in cases (116/150, 77.3%) compared with all controls (81/134, 60.4%; *P* = 0.002) and healthy controls (55/90, 61.1%; *P* = 0.007). Compared to healthy controls, cases reported higher rates of chronic illnesses (97/150, 64.7% vs. 47/90, 52.2%; *P* = 0.06), hypertension (66/150 or 44% vs. 31/82 or 37.8%; *P* = 0.29), and dyslipidaemia (41/150 or 27.3% vs. 17/90 or 18.9%; *P* = 0.14). While most cases and controls had a normal BMI range, a moderately higher percentage of cases were obese (6.8%) compared with controls (3% for all controls; 1.1% for healthy controls).

**Table 1.** Baseline characteristics of cases vs. all controls and healthy controls

Characteristics	Cases, *N* = 150	All controls^a^, *N* = 134	*P*-value	Healthy controls, *N* = 90	*P*-value
Age					
40–64 years	124 (82.7%)	111 (82.8%)	0.97	77 (85.6%)	0.56
≥65 years	26 (17.3%)	23 (17.2%)		13 (14.4%)	
Male	146 (97.3%)	127 (94.8%)	0.26	86 (95.6%)	0.46
Marital status					
Never married/divorced/separated/widow	4 (2.7%)	0 (0%)	0.06	0 (0%)	0.12
Married	145 (97.3%)	134 (100%)		90 (100%)	
Family size category					
<5	112 (74.7%)	97 (72.4%)	0.66	67 (74.4%)	0.97
≥5	38 (25.3%)	37 (27.6%)		23 (25.6%)	
Location					
Urban	70 (46.7%)	53 (39.6%)	0.23	35 (38.9%)	0.24
Rural	80 (53.3%)	81 (60.4%)		55 (61.1%)	
Occupation					
Unemployed/house maker	16 (10.7%)	26 (19.4%)	0.29	11 (12.2%)	0.88
Service	16 (10.7%)	11 (8.2%)		6 (6.7%)	
Business	65 (43.3%)	50 (37.3%)		39 (43.3%)	
Day labourer/agriculture	19 (12.7%)	19 (14.2%)		12 (13.3%)	
Others	34 (22.7%)	28 (20.9%)		22 (24.4%)	
Education					
<primary level	60 (41.1%)	49 (38.3%)	0.69	26 (31%)	0.24
Primary to HSC	80 (54.8%)	71 (55.5%)		52 (61.9%)	
Completed graduation	6 (4.1%)	8 (6.3%)		6 (7.1%)	
Ever used tobacco	116 (77.3%)	81 (60.4%)	0.002	55 (61.1%)	0.007
Participants engaging in moderate physical activity	80 (53.3%)	75 (56.0%)	0.66	54 (60.0%)	0.31
Family history of cardiovascular diseases	107 (71.3%)	92 (68.7%)	0.6	71 (78.9%)	0.19
History of at least one chronic illness	97 (64.7%)	80 (59.7%)	0.39	47 (52.2%)	0.06
Hypertension^b^	66 (44%)	48 (41.7%)	0.6	31 (37.8%)	0.29
Diabetes^b^	29 (19.3%)	26 (22.6%)	0.5	18 (21.9%)	0.63
High blood cholesterol	41 (27.3%)	27 (20.1%)	0.16	17 (18.9%)	0.14
BMI category					
<18.5	18 (12.2%)	12 (9.1%)	0.34	5 (5.6%)	0.06
18.5–24.9	78 (52.7%)	80 (60.6%)		55 (61.8%)	
25–29.9	42 (28.4%)	36 (27.3%)		28 (31.5%)	
>30	10 (6.8%)	4 (3.0%)		1 (1.1%)	

a Includes both cardiac and healthy controls.

b A total of 33 cardiac control and 82 healthy control participants had information available.

### Respiratory viruses and influenza among cases and controls

Of the 284 participants, 19 (6.69%) tested positive for any respiratory virus, including 8/150 (5.3%) cases and 11/134 (8.2%) controls, and the difference was not statistically significant (*P* > 0.05) (Table 2). Seven cases of AMI (4.7%) and five controls (3.7%) had laboratory-confirmed influenza (Table 2). Of all influenza strains detected among AMI cases, five were A/H3 and two were A/H1N1pdm09. None of the AMI cases were positive for influenza B. The rate of laboratory-confirmed influenza was not significantly different between cases and cardiac controls, with seven cases (4.7%) and two controls (4.5%) positive for influenza, respectively (*P* = 0.97). Adenovirus was the only other respiratory virus detected in a single case of AMI. There was no significant difference (*P* = 0.62) between the rates of influenza in the cases (7/150, 4.7%) compared with healthy controls (3/90, 3.3%) (Table 2).

**Table 2.** Laboratory-confirmed influenza and other respiratory viruses among cases vs. all controls and healthy controls

Laboratory-confirmed influenza and other respiratory viruses	Cases, *N* = 150	All controls^a^, *N* = 134	*P*-value	Healthy controls, *N* = 90	*P*-value
All respiratory viruses including influenza	8 (5.3%)	11 (8.2%)	0.74	5 (5.5%)	0.24
All influenza types	7 (4.7%)	5 (3.7%)	0.70	3 (3.3%)	0.62
All influenza A	7 (4.7%)	5 (3.7%)	0.70	3 (3.3%)	0.62
A/H3	5 (3.3%)	3 (2.2%)	0.58	1 (1.1%)	0.29
A/H1N1pdm09	2 (1.3%)	2 (1.5%)	0.91	2 (2.2%)	0.60
Influenza B	0 (0%)	0 (0%)	–	0 (0%)	–
Other respiratory viruses	1 (0.7%)	6 (4.5%)	0.04	2 (2.2%)	0.29
Human metapneumovirus	0 (0%)	1 (0.7%)	–	0 (0%)	–
Respiratory syncytial virus	0 (0%)	1 (0.7%)	–	1 (1.1%)	–
Adenovirus	1 (0.7%)	4 (3%)	–	1 (1.1%)	–

a Includes both cardiac and healthy controls.

### Comparative analysis of recent clinical respiratory infections among cases and controls

**Figure 1.**
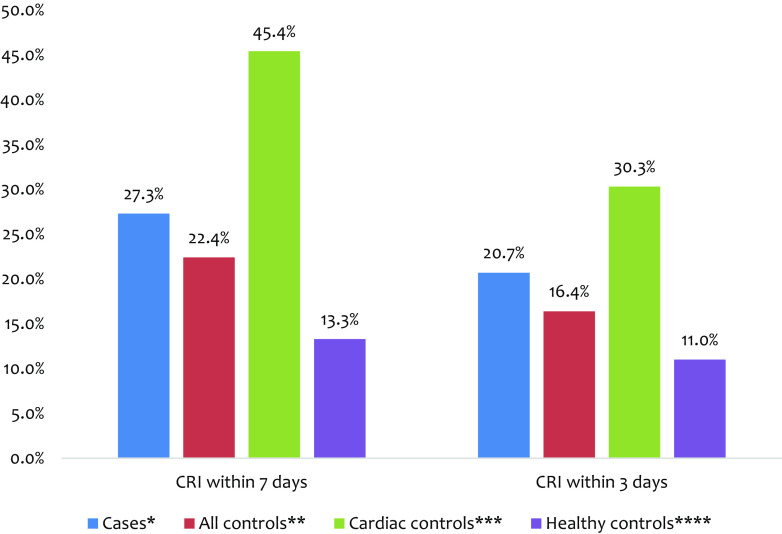
Clinical respiratory illness rates among cases and controls. *150 cases. **134 all controls. ***44 cardiac controls. ****90 healthy controls.

Figure 1 illustrates the percentages of recent CRI among various groups, comprising AMI cases, all controls, cardiac controls, and healthy controls. A total of 41 of 150 (27.3%) AMI cases had recent CRI within a week before the onset of AMI, which was higher than the rates among all controls (30/134, 22.4%) and healthy controls (12/90, 13.3%). In contrast, the proportion of recent CRI was significantly higher among cardiac controls, with 20 of 44 (45.5%) reporting recent CRI compared with cases (27.3%). Furthermore, the comparison of CRI proportions with onset within the past three days between cases and controls revealed a similar trend.

### Association of respiratory infections with AMI: Logistic regression analysis

In both univariate (OR: 2.43; 95% CI: 1.17–5.04) and multivariate (aOR: 2.21; 95% CI: 1.05–4.6) analyses (Table 3), cases had significantly higher odds of reporting CRI within the past week compared with healthy controls. However, this association was no longer significant when cases were compared to all controls (cardiac plus healthy controls; aOR: 1.19, 95% CI: 0.65–2.18) (Table 3). While cases had higher odds of having laboratory-confirmed influenza compared with all controls (aOR: 1.55; 95% CI: 0.42–5.69) and healthy controls (aOR: 2.59; 95% CI: 0.50–13.36), the associations did not reach statistical significance after adjusting for other factors (Table 4).

**Table 3.** Association of recent clinical respiratory illness with acute myocardial infarction in cases compared with all controls and healthy controls

Variable	Cases, N = 150	All controls^a^, N = 134	aOR (95% CI)^b^	Healthy controls, N = 90	aOR (95% CI)^b^
Recent clinical respiratory illness					
No	109 (72.7%)	104 (77.6%)	Ref.	78 (86.7%)	Ref.
Yes	41 (27.3%)	30 (22.4%)	1.19 (0.65–2.18)	12 (13.3%)	2.21 (1.05–4.6)
Age					
40–64 years	124 (82.7%)	111 (82.8%)	Ref.	77 (85.6%)	Ref.
≥65 years	26 (17.3%)	23 (17.2%)	0.69 (0.34–1.41)	13 (14.4%)	0.97 (0.35–2.65)
Gender					
Male	146 (97.3%)	127 (94.8%)	1.96 (0.43–8.94)	86 (95.6%)	2.97 (0.54–16.42)
Female	4 (2.7%)	7 (5.2%)	Ref.	4 (4.4%)	Ref.
Tobacco use					
No	34 (22.7%)	53 (39.6%)	Ref.	35 (38.9%)	Ref.
Yes	116 (77.3%)	81 (60.4%)	1.71 (0.89–3.28)	55 (61.1%)	1.96 (1.01–3.79)
Moderate physical activity					
No	70 (46.7%)	59 (44.0%)	Ref.	36 (40.0%)	Ref.
Yes	80 (53.3%)	75 (56.0%)	0.80 (0.45–1.44)	54 (60.0%)	0.51 (0.24–1.09)
Hypertension					
No	84 (56.0%)	69 (59%)	Ref.	53 (63.1%)	Ref.
Yes	66 (44.0%)	48 (41.0%)	1.14 (0.67–1.93)	31 (36.9%)	1.42 (0.77–2.6)
Diabetes					
No	121 (80.7%)	89 (77.4%)	Ref.	64 (78.0%)	Ref.
Yes	29 (19.3%)	26 (22.6%)	0.87 (0.46–1.64)	18 (22%)	1.01 (0.5–2.07)
High blood cholesterol					
Normal	109 (72.7%)	107 (79.9%)	Ref.	73 (81.1%)	Ref.
High	41 (27.3%)	27 (20.1%)	1.36 (0.68–2.69)	17 (18.9%)	2.08 (0.82–5.30)
BMI					
Under 18.5	18 (12.2%)	12 (9.1%)	Ref.	5 (5.6%)	Ref.
18.5 to 24.9	78 (52.7%)	80 (60.6%)	0.57 (0.17–1.72)	55 (61.8%)	0.80 (0.19–3.32)
25.0 to 29.9	42 (28.4%)	36 (27.3%)	0.77 (0.23–2.57)	28 (31.5%)	0.90 (0.19–4.18)
Over 30	10 (6.8%)	4 (3.0%)	0.97 (0.20–4.76)	1 (1.1%)	4.80 (0.38–60.58)

a Includes both cardiac and healthy controls.

b Variable adjusted for in the final model: age, gender, tobacco use status, physical activity level, history of hypertension, diabetes, high blood cholesterol, and body mass index (BMI).

**Table 4.** Association of influenza with acute myocardial infarction in cases compared with all controls and healthy controls

Variable	Cases, *N* = 150	All controls^a^, *N* = 134	aOR (95% CI)^b^	Healthy controls, *N* = 90	aOR (95% CI)^b^
Influenza					
No	143 (95.3%)	129 (96.3%)	Ref.	87 (96.7%)	Ref.
Yes	7 (4.7%)	5 (3.7%)	1.55 (0.42–5.69)	3 (3.3%)	2.59 (0.50–13.36)
Age					
40–64 years	124 (82.7%)	111 (82.8%)	Ref.	77 (85.6%)	Ref.
≥65 years	26 (17.3%)	23 (17.2%)	1.16 (0.60–2.24)	13 (14.4%)	1.35 (0.59–2.99)
Gender					
Male	146 (97.3%)	127 (94.8%)	1.74 (0.40–7.56)	86 (95.6%)	1.63 (0.34–7.81)
Female	4 (2.7%)	7 (5.2%)	Ref.	4 (4.4%)	Ref.
Tobacco use					
No	34 (22.7%)	53 (39.6%)	Ref.	35 (38.9%)	Ref.
Yes	116 (77.3%)	81 (60.4%)	2.05 (1.16–3.63)	55 (61.1%)	1.87 (0.98–3.58)
Moderate physical activity					
No	70 (46.7%)	59 (44.0%)	Ref.	36 (40.0%)	Ref.
Yes	80 (53.3%)	75 (56.0%)	0.85 (0.51–1.43)	54 (60.0%)	0.66 (0.37–1.20)
Hypertension					
No	84 (56.0%)	69 (59%)	Ref.	53 (63.1%)	Ref.
Yes	66 (44.0%)	48 (41.0%)	1.14 (0.67–1.94)	31 (36.9%)	1.44 (0.79–2.62)
Diabetes					
No	121 (80.7%)	89 (77.4%)	Ref.	64 (78.0%)	Ref.
Yes	29 (19.3%)	26 (22.6%)	0.88 (0.47–1.65)	18 (22%)	1.07 (0.53–2.17)
High blood cholesterol					
Normal	109 (72.7%)	107 (79.9%)	Ref.	73 (81.1%)	Ref.
High	41 (27.3%)	27 (20.1%)	1.53 (0.83–2.83)	17 (18.9%)	2.00 (0.98–4.11)
BMI					
Under 18.5	18 (12.2%)	12 (9.1%)	Ref.	5 (5.6%)	Ref.
18.5 to 24.9	78 (52.7%)	80 (60.6%)	0.39 (0.14–1.07)	55 (61.8%)	0.35 (0.11–1.14)
25.0 to 29.9	42 (28.4%)	36 (27.3%)	0.49 (0.16–1.45)	28 (31.5%)	0.38 (0.11–1.35)
Over 30	10 (6.8%)	4 (3.0%)	0.97 (0.21–4.44)	1 (1.1%)	2.68 (0.25–28.36)

a Includes both cardiac and healthy controls.

b Variable adjusted for in the final model: age, gender, tobacco use status, physical activity level, history of hypertension, diabetes, high blood cholesterol, and body mass index (BMI).

### Comparison of demographic characteristics between cases and cardiac controls

The comparison of AMI cases with cardiac controls showed that most patients in both groups were in the age range of 40–64 years (82.7% cases, 77.3% controls) and were predominantly male (97.3% cases, 93.2% controls). Most patients were married and resided in family units of <5 members. Urban and rural distributions were fairly even in both groups. The occupational status was significantly different between AMI cases and cardiac controls (*P* = 0.003). There were more unemployed among the cardiac control group than the AMI cases (27.3% vs. 10.7%), and 43.3% of AMI patients compared with 25% of cardiac controls were in business, with similar education levels across both groups. The use of tobacco was notably higher in the case group (77.3% versus 59.1% in cardiac controls; *P* = 0.02), and moderate physical activity was practised similarly by both groups. A comparison of clinical characteristics among AMI cases vs. cardiac controls showed that a higher proportion of cases had a family history of CVDs (60.0%) compared with controls (47.7%). A considerable proportion of both groups reported at least one chronic illness (64.7% cases and 75.0% controls). Hypertension was found in 44% of the cases and 38.6% of the controls, while the prevalence of diabetes was similar in both groups (19.3% cases and 18.2% controls). High blood cholesterol was slightly more common in cases (13.3%) compared with controls (11.4%). The BMI status was similar across both groups.

### Comparative analysis of respiratory infection rates between cardiac and healthy control participants

We also compared CRI and influenza rates between cardiac and healthy control participants. In univariate analysis, the proportion of individuals with CRI was significantly higher in cardiac controls (40.9%) compared with healthy controls (21.1%). The unadjusted OR for CRI among cardiac controls vs. healthy controls was 2.59 (95% CI: 1.18–5.68). After adjusting for potential confounders, the association remained, although the effect size (aOR) decreased slightly to 2.18 (95% CI: 0.89–5.31), which was, however, not statistically significant (*P* = 0.09). There was no significant difference in influenza prevalence between the two groups, with 4.5% of cardiac controls and 3.3% of healthy controls testing positive for influenza (OR: 1.38; 95% CI: 0.22–8.58). After adjusting for potential confounders, this association remained non-significant, with the effect size (aOR) increasing to 2.48 (95% CI: 0.13–48.63).

## Discussions

To the best of our knowledge, this study was the first to investigate the association between recent CRIs and laboratory-confirmed influenza with AMI, during an annual influenza season in Bangladesh. In the current study, the AMI patients were about twice as likely as healthy individuals to report a recent CRI during the past week. Cardiac controls had the highest frequency of CRI. Although the frequency of the occurrence of laboratory-confirmed influenza was also higher among AMI cases compared with all controls, the study may have been underpowered to detect a significant difference. These results suggest that recent acute respiratory illnesses may trigger adverse acute CV events such as AMI or other cardiac events, during the influenza season in Bangladesh. Our findings suggest that there may be a window of opportunity to prevent AMI among the Bangladeshi population by advocating preventive measures to control influenza and other respiratory infections in elderly and high-risk individuals, such as improving infection prevention and control practices and enhancing influenza vaccine awareness.

Previous case–control studies have found an association between recent respiratory illnesses and AMI in elderly people [22, 26-28]; however, there were notable differences in control selection, influenza season timing, and sample size across these studies. MacIntyre et al. [22] found a significant association between recent acute respiratory tract infection and AMI cases, but only in univariate analysis (OR: 1.98; 95% CI: 1.2 to 3.3). In comparison with ours, unmatched orthopaedic or ophthalmic outpatient controls were used in this study, who were older than our healthy controls, potentially influencing the findings. We investigated a single 2018 influenza season, while MacIntyre et al. covered three seasons (2008, 2009, and 2010), utilizing a more extensive sample size. Studying multiple seasons provides a more comprehensive understanding of the association and accounts for seasonal variation and different circulating influenza strains. Our single-season study may have missed some of this variability, leading to a less complete understanding of the relationship between recent respiratory respiratory illness with with AMI. It is crucial to account for the number and variability of influenza seasons studied when interpreting findings on this relationship [29–31]. Additionally, the criteria utilized by MacIntyre et al. [22] to define recent respiratory illness required the presence of both respiratory and systemic symptoms within the past week of AMI. In contrast, our criteria did not include systemic symptoms, which could potentially decrease clinical infection rates in our study.

Warren et al. [26] also found a similar association between recent acute respiratory illness and AMI, using controls from non-cardiac patients. However, the study occurred during the 2009 influenza pandemic with a different case definition and smaller sample size, resulting in a non-significant association. The timing of studies is crucial, as pandemics can provide different insights due to variations in virulence, transmission, and prevalence of the influenza virus, affecting generalizability. Contextual factors such as pandemics may influence the association between influenza, other respiratory viruses, and AMI due to varying levels of awareness and preparedness. Like MacIntyre et al., Warren et al. [26] defined recent respiratory illness as the presence of both respiratory and systemic symptoms within a month of AMI. Variances in estimates may also be attributed to the fact that some studies were exclusively conducted during influenza seasons [22, 26], while others encompassed both influenza and non-influenza seasons [27], as the incidence of respiratory illness is known to fluctuate throughout the year.

A few case–control studies utilizing general practice (GP) data also reported the association between ARI and AMI [27, 32]. The advantage of utilizing a GP database is that it allows for the investigation of a large number of participants and provides access to reliable, patient-specific data for the study design. Moreover, the selection of controls from the same GP as the cases reduced the potential for confounding factors, such as differences in healthcare access or socio-economic status. This method of control selection helps to reduce the potential for confounding factors and allows for a more accurate assessment of the association between exposure and outcome.

In general, AMI cases were twice as likely as controls to report a recent respiratory illness within 7 days before AMI onset. This association has weakened over time since AMI onset [22, 26, 27]. Meta-analyses of self-controlled case-series studies showed that AMI risk significantly increased within three days of recent influenza-related respiratory illness (pooled incidence rate ratio: 5.79; 95% CI: 3.59–9.38) [7], while that of the case–control studies showed a doubled risk (pooled odds ratio: 2.01; 95% CI: 1.47–2.76) [8]. These findings emphasize the need for early detection and management of respiratory illness in patients with cardiac disease.

The lack of a significant association between laboratory-confirmed influenza and AMI in our study could be due to low influenza positivity in both cases and controls. In previous studies, influenza detection rates among AMI patients varied widely (14% to 86.3%), varying by testing method (serology vs. PCR), influenza strain distribution and virulence, and the number of seasons studied [8]. The WHO recommends RT-PCR testing of respiratory swabs within 10 days of symptom onset to maximize detection before viral shedding ceases [33]. However, the exact timeline of AMI onset after influenza infection is uncertain, and viral shedding may be undetectable before AMI patients are swabbed [22, 26, 34]. Generally, most viral shedding occurs within 2–3 days of respiratory infection onset [35]. Therefore, in our study, we tested patients who provided specimens within 72 h of AMI symptom onset. Using baseline serology [34] and analysis of paired sera [22] for IgG, or baseline serology for IgA [26], may augment the detection of additional influenza cases with or without PCR.

In our study, the identified influenza subtypes among AMI patients matched the strains circulating in Bangladesh during the 2018 influenza season, as reported by the national surveillance programme [31]. This suggests that surveillance may effectively predict and monitor the impact of influenza on CV health. Our PCR-confirmed influenza rate among AMI cases was higher than in some other studies [22, 26]. Although our study did not show a clear association between influenza and AMI, few case–control studies have demonstrated a direct link, possibly due to the low influenza incidence in age groups prone to AMI [34]. However, many studies have reported the effectiveness of influenza vaccines in preventing AMI in these age groups [8, 22], supporting influenza’s association with AMI.

AMI is primarily caused by a blocked coronary artery due to atherothrombosis, leading to heart muscle damage. This can result from a ruptured atherosclerotic lesion triggered by factors such as viral infections, smoking, alcohol, hypertension, exertion, or stress [36–39]. The immune system’s role in AMI is crucial, as a pro-inflammatory response can accelerate atherosclerosis, causing events such as AMI. Influenza can increase inflammation and blood clotting, destabilizing plaques and causing AMI [40, 41]. Individual differences in inflammation intensity may affect AMI risk after triggers such as influenza [42].

Several observational studies [8, 22, 43] and small-scale [44, 45] and large-scale [9, 46] randomized clinical trials reported the protective efficacy of the influenza vaccine against AMI. An animal study showed influenza vaccine-stabilized atherosclerotic plaque by promoting an anti-inflammatory atheroprotective immune response [47]. Additionally, the influenza vaccine has been shown to blunt pro-inflammatory and enhance anti-inflammatory mediators after coronary artery bypass surgery [48].

We acknowledge several limitations in the current study. First, we included cardiac controls in the initial phase of the study, but later recruited healthy controls to minimize the chance of selection bias due to the misclassification of cardiac controls. Switching to seemingly healthy controls during the study broadened the scope of the research question but also potentially introduced bias into the data due to the significant differences within the control group. The selection of healthy controls from visitors to the unit might have led to an overmatching of exposure due to the household transmission of common respiratory infections. Cardiac controls might have been susceptible to CRI or influenza-related cardiac crises and hospitalizations, leading to a higher occurrence of CRI or influenza in controls than expected. This problem is often seen with hospital-based disease controls [49], as, essentially, influenza can initiate other cardiac issues, in vulnerable individuals. Influenza can raise the risk of cardiac complications, including acute heart failure [50] and arrhythmias, leading to sudden cardiac arrest [51], due to increased cardiac stress during respiratory illness and potential direct infection of the heart muscle, causing myocarditis and further issues. However, incorporating both cardiac and healthy controls enhanced the current study’s comparability by allowing a more balanced representation of the population and mitigating potential confounding variables. Moreover, the study’s generalizability is heightened with healthy controls representing the general population, facilitating the extension of findings to a broader demographic. Utilizing a mix of controls augmented the study’s statistical power due to a larger sample size, enhancing the detection of significant differences and precision of estimates. Lastly, potential confounding factors specific to either group can be addressed, thus improving the study’s validity and reliability. Yet, one of the limitations of any case–control study including the current study is the potential for unknown residual confounding variables, not accounted for during the study implementation, which may obscure the true relationship between the exposure and outcome, making it difficult to draw accurate conclusions. We have, however, conducted an age- and gender-matched case–control study to enable us to control for these potential confounders. In addition, the current study’s multivariable analysis approach enabled us to control for confounding variables more effectively. A second limitation is that our study excluded systemic symptoms from the respiratory case definition, which may have underestimated the true prevalence of respiratory illnesses in our study population. We cannot also exclude the possibility of misdiagnosing the prodromal state preceding AMI. Third, the research was conducted during a single influenza season rather than spanning multiple years and in a single-study hospital as opposed to multiple study sites that may limit the generalizability of the findings across different years or seasons and throughout the whole country. This might have also potentially limited the optimal sample size required to accurately identify the associations. A fourth limitation of the current study is that we did not use paired serum analysis for influenza detection to complement PCR results, which may have missed some influenza cases, leading to an underestimation of the association between laboratory-confirmed influenza and AMI. Concerning the sample size, the number of participants enrolled in testing PCR-confirmed influenza was insufficient for detecting an association. This limitation became more pronounced considering the notably low prevalence of influenza, in both the case and control groups in the current study. We propose that future studies address it by increasing the sample size, particularly given the limited number of influenza cases among the participants. Lastly, we aimed for a 1:1 recruitment ratio of controls to AMI cases. However, unexpected challenges arose, including a high prevalence of ischaemic complications among inpatients, making it difficult to find suitable cardiac controls. Further complicating recruitment was logistical hurdles such as eligible healthy controls declining participation or not meeting our criteria. Additionally, we faced budgetary constraints that limited our capacity to conduct the necessary number of PCR tests for influenza. These practical constraints resulted in recruiting only 134 controls instead of the planned equal number of 150 cases.

## Conclusions

Acute respiratory illnesses may increase the risk of AMI among high-risk individuals during the influenza season in Bangladesh. Patients and providers should be aware of the potential risk of AMI onset after an acute respiratory infection and may consider this during clinical evaluation. Larger-scale studies over multiple influenza seasons may provide more accurate estimates of the associations. Given the high burden of both adverse CV events, evidence of circulating seasonal influenza among hospitalized patients, and underutilization of influenza vaccines in Bangladesh, advocating infection prevention and control practices and influenza vaccination could serve as a cost-effective strategy to manage the health risk of influenza and other respiratory infections in the country.

## Data Availability

Data generated during the study are subject to the data access policy of ICDDR,B and are available from ICDDR,B’s research administration on reasonable request through the corresponding author.

## Abbreviations

AMI: Acute myocardial infarction
aOR: Adjusted odds ratio
BMI: Body mass index
CCU: Coronary care units
CRI: Clinical respiratory illnesses
ECG: Electrocardiogram
HMPV: Human metapneumovirus
HPAV: Human parainfluenza virus
IAMI: Influenza Vaccination After Myocardial Infarction
ICDDR,B: International Centre for Diarrheal Disease Research, Bangladesh
IRR: Incidence Rate Ratio
MACE: Major adverse cardiovascular events
NICVD: National Institute of Cardiovascular Diseases
OR: Odds ratio
RSV: Respiratory syncytial virus
ST: S and T waves of an electrocardiogram
qRT-PCR: Quantitative real-time reverse transcription polymerase chain reaction
VIF: Variance inflation factor

## Patient and public involvement

Patients and/or the public were not involved in the design, conduct, reporting, or dissemination plans of this research.
